# Human GBP1 does not localize to pathogen vacuoles but restricts *Toxoplasma gondii*

**DOI:** 10.1111/cmi.12579

**Published:** 2016-03-10

**Authors:** Ashleigh C. Johnston, Anthony Piro, Barbara Clough, Malvin Siew, Sebastian Virreira Winter, Jörn Coers, Eva‐Maria Frickel

**Affiliations:** ^1^ Host-Toxoplasma Interaction LaboratoryThe Francis Crick InstituteMill Hill LaboratoryLondonNW7 1AAUnited Kingdom; ^2^ Departments of Molecular Genetics and Microbiology and ImmunologyDuke University Medical CenterDurhamNC27710USA; ^3^ Max Planck Institute for Infection BiologyCharitéplatz 110117BerlinGermany

## Abstract

Guanylate binding proteins (GBPs) are a family of large interferon‐inducible GTPases that are transcriptionally upregulated upon infection with intracellular pathogens. Murine GBPs (mGBPs) including mGBP1 and 2 localize to and disrupt pathogen‐containing vacuoles (PVs) resulting in the cell‐autonomous clearing or innate immune detection of PV‐resident pathogens. Human GBPs (hGBPs) are known to exert antiviral host defense and activate the NLRP3 inflammasome, but it is unclear whether hGBPs can directly recognize and control intravacuolar pathogens. Here, we report that endogenous or ectopically expressed hGBP1 fails to associate with PVs formed in human cells by the bacterial pathogens *Chlamydia trachomatis* or *Salmonella typhimurium* or the protozoan pathogen *Toxoplasma gondii*. While we find that hGBP1 expression has no discernible effect on intracellular replication of *C*. *trachomatis* and *S*. *typhimurium*, we observed enhanced early *Toxoplasma* replication in CRISPR hGBP1‐deleted human epithelial cells. We thus identified a novel role for hGBP1 in cell‐autonomous immunity that is independent of PV translocation, as observed for mGBPs. This study highlights fundamental differences between human and murine GBPs and underlines the need to study the functions of GBPs at cellular locations away from PVs.

## Introduction

The cytokine Interferon gamma (IFNγ) is an important mediator of host response against an array of intracellular pathogens (MacMicking, 2012). Such infections include the apicomplexan parasite *Toxoplasma gondii*, in which case IFNγ drives effector mechanisms to eliminate the fast replicating acute phase tachyzoite stage. The intravacuolar bacteria *Chlamydia trachomatis* and *Salmonella typhimurium* are likewise targeted by IFNγ‐driven host responses in the acute stages of infection, resulting in *C*. *trachomatis* reticulate bodies forming an aberrant non‐dividing form, and in *S*. *typhimurium* clearance (Kazar *et al*., 1971; Pie *et al*., 1997). In response to infection, IFNγ upregulates a vast number of proteins, with a family of large GTPases, the Guanylate Binding Proteins (GBPs), being among the most highly induced (Cheng *et al*., 1983). GBPs have been studied *in vitro* or in murine models and are important in immune activation and restricting intracellular pathogens, including viruses, bacteria and protozoan parasites (MacMicking, 2012).

In mice, mGBPs accumulate around pathogen‐containing vacuoles (PV) of intracellular pathogens such as *Toxoplasma* (Degrandi *et al*., 2007; Virreira Winter *et al*., 2011; Haldar *et al*., 2013; Selleck *et al*., 2013), *C*. *trachomatis* (Coers *et al*., 2008; Haldar *et al*., 2013), *Mycobacterium bovis* BCG (Kim *et al*., 2011) and *S*. *typhimurium* (Meunier *et al*., 2014). At PVs mGBPs act cooperatively to assemble host defense responses that include an oxidative burst, the delivery of antimicrobial peptides and the induction of autophagy (Kim *et al*., 2011). Additionally, mGBPs promote the disintegration of *Salmonella*‐containing vacuoles thereby exposing bacteria to the cytosol where they can activate the cytosolic LPS sensor caspase‐11 (Meunier *et al*., 2014). Rapid activation of caspase‐11 in response to infections with *Legionella pneumophila* or *Chlamydia muridarum* requires additional lysis‐independent function of mGBPs that are poorly characterized (Pilla *et al*., 2014; Finethy *et al*., 2015). In addition to their association with PVs mGBPs also co‐localize with the cytosolic bacterial pathogens *Listeria monocytogenes* and *Francisella novicida* (Kim *et al*., 2011; Man *et al*., 2015; Meunier *et al*., 2015). The association of mGBPs with *F*. *novicida* prompts bacteriolysis and the activation of the cytosolic DNA sensor AIM2 (Man *et al*., 2015; Meunier *et al*., 2015).

Human GBPs are much less well understood, and their functional significance remains largely unknown. hGBP5 promotes NLRP3 inflammasome assembly and consequently IL‐1β production in response to LPS and nigericin (Shenoy *et al*., 2012). hGBP1 can mediate the inhibition of endothelial cell proliferation (Guenzi *et al*., 2001) and has been shown to exhibit anti‐viral properties. When hGBP1 expression is silenced using small hairpin RNAs, Dengue virus burden increases (Pan *et al*., 2012). hGBP1 also mediates an antiviral effect against vesicular stomatitis virus and encephalomyocarditis virus when overexpressed in HeLa cells (Anderson *et al*., 1999). While these studies indicate that human GBPs have relevance during pathogenic infection, we do not understand which microbes they target and how the endogenous proteins act.

The structure and biochemical properties of GBPs have been studied in detail, with the structure of hGBP1 revealing a globular GTPase domain connected to an arm‐like extension, and revealing the very fast GTP hydrolysis rate of 95 min^−1^ (Prakash *et al*., 2000). Some hGBPs also have the ability to perform two consecutive hydrolysis steps from GTP to GMP (Schwemmle and Staeheli, 1994). hGBP 1, 2 and 5 contain a ‘CaaX’ prenylation motif at their C‐terminus, implying a capacity to target membranes.

In agreement with their biophysical properties and observations made for mGBPs, hGBPs have been postulated to recognize PVs formed by type II *Toxoplasma* vacuoles as well as *C*. *trachomatis* PVs, also known as inclusions (Tietzel *et al*., 2009; Al‐Zeer *et al*., 2013; Ohshima *et al*., 2014). It has been reported that interference with hGBP1 and 2 expression in IFNγ‐primed cells led to increased *C*. *trachomatis* inclusions size, indicating better growth of the bacteria in absence of these proteins (Tietzel *et al*., 2009). Many of these studies have relied on heterologously expressed proteins or an antibody recognizing several GBP family members. A recent study on *Toxoplasma* postulates no effect on parasite restriction by hGBPs (Ohshima *et al*., 2014). It thus remains unclear which, if any, hGBPs target *Toxoplasma* PVs, and whether endogenous hGBPs can target and restrict *C*. *trachomatis*.

Here, we demonstrate that hGBP1, in contrast to its closest murine orthologues mGBP1 and 2 fails to recognize PV formed by *C*. *trachomatis*, *S*. *typhimurium* or *Toxoplasma* in human epithelial cells. Our data indicate that hGBP1 is not essential for the execution of cell‐autonomous control of the replication of these pathogens in unprimed and IFNγ‐primed human epithelial cells. However, in a similar manner to mGBPs, hGBP1 is able to restrict *Toxoplasma* early after host cell infection. This restriction cannot be attributed to an invasion mechanism, but rather hGBP1 is responsible for delaying the onset of parasite replication. GBPs have only been reported to play a role in cell‐autonomous control of infections if they accumulated around the pathogen‐containing vacuole. Here we show that this is not the case for hGBP1. We thus define a novel role for hGBP1 with its capacity to restrict *Toxoplasma* in early infection without targeting to the PV.

## Results

### hGBP1 does not localize to intracellular pathogen vacuoles in epithelial cells

Several mGBPs recruit to PVs in an IFNγ‐dependent manner, disrupt PV integrity and facilitate pathogen destruction. We explored whether hGBP1 was able to target intracellular vacuoles formed by the bacterial pathogens *C*. *trachomatis* and *S*. *typhimurium* or the parasite *Toxoplasma*. Human GBP1 is the closest orthologue of mGBP2 previously shown to associate with vacuolar *C*. *trachomatis*, *S*. *typhimurium* and *Toxoplasma* in murine fibroblasts, epithelial cells, macrophages and spleen tissue (Degrandi *et al*., 2007; Tietzel *et al*., 2009; Virreira Winter *et al*., 2011; Al‐Zeer *et al*., 2013; Haldar *et al*., 2013; Selleck *et al*., 2013; Meunier *et al*., 2014). To this end, we produced a specific peptide antibody that could distinguish hGBP1 from all other hGBPs by immunoblot, and additionally a pan‐hGBP antibody that recognized hGBPs 1, 2, 3 and 5 (Fig. S1A). While we could not overexpress hGBP4, this family member is least identical to hGBP1 so we are confident the antibody will not cross react. We show that A549 cells express GBPs including hGBP1 at steady state level and further upregulate the protein in response to IFNγ (Fig. S1B).

To further characterize this newly generated anti‐hGBP1 antibody, we employed CRISPR/Cas9 technology to generate a lung epithelial A549‐derived cell line lacking hGBP1 expression. A region 5′ to the GTPase domain of *Gbp1* was targeted for disruption, and deletion was confirmed by sequencing and immunoblot (Fig. S1C and D). The cells were further analysed by the pan‐hGBP antibody, demonstrating that other hGBPs are still intact with normal protein expression (Fig. S1D). Using anti‐hGBP1 for immunofluorescence staining we found that both naïve and IFNγ‐primed and ΔhGBP1 cells exhibited low background immunofluorescence intensity. In contrast, parental A549 cells showed a robust increase in the anti‐hGBP1 immunofluorescence signal when primed with IFNγ (Fig. S1E), demonstrating that anti‐hGBP1 specifically detects endogenous hGBP1 *in situ*.

**Figure 1:**
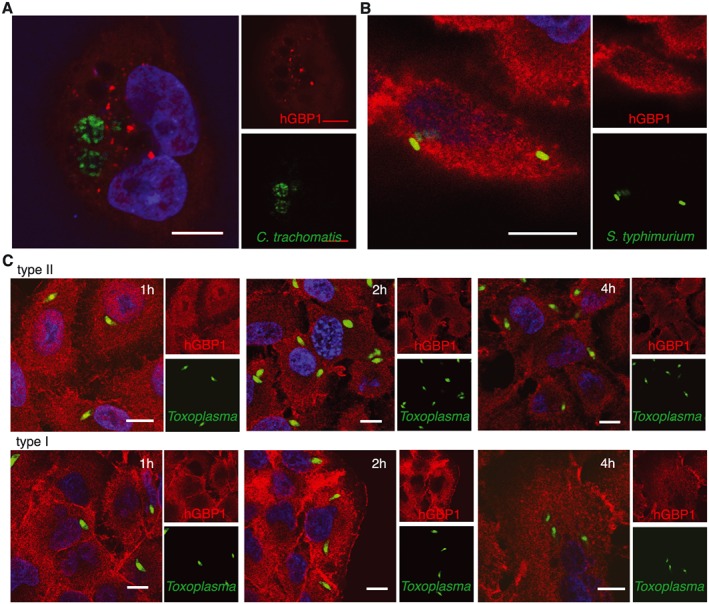
hGBP1 does not localize to the intracellular pathogen vacuole. A. Immunofluorescent confocal image of *C*. *trachomatis* vacuoles 20hpi in mCherry‐hGBP1 expressing A549 cells primed with 200 U/ml IFNγ. *N* = 2. B. Immunofluorescent confocal image of *S*. *typhimurium* vacuoles 4hpi in A549 cells primed with 50 U/ml IFNγ stained for endogenous hGBP1. *N* = 3. C. Immunofluorescent confocal image of *Toxoplasma* vacuoles in A549 cells primed with 10 U/ml IFNγ at the indicated time points post infection. *N* = 3. All scale bars 10 µm.

Having confirmed the specificity of the anti‐hGBP1 antibody we asked whether hGBP1 associated with PVs. To do so, we infected IFNγ‐primed A549 cells with three representative vacuolar pathogens. Immunofluorescent microscopy showed that hGBP1 does not recruit around the PVs formed by *C*. *trachomatis* (Fig. 1A), *S*. *typhimurium* (Fig. 1B) or *Toxoplasma* (Fig. 1C). This lack of recruitment was observed at all time points tested (data not shown) and not affected by the absence of presence of IFNγ priming (Fig. S2A to C).

In order to associate with membranes hGBPs first transition into an active GTP‐bound state and following hydrolysis hGBPs in the GDP‐bound state form tetramers that can attach to membranes (Syguda *et al*., 2012). We therefore considered that the anti‐hGBP1 antibody failed to detect hGBP1 in the active, membrane‐associated state. To test for this, we ectopically expressed hGBP1 N‐terminally fused to mCherry. We observed that mCherry‐hGBP1 heterologously expressed in mouse embryonic fibroblast (MEFs) localized to *Toxoplasma* PVs (Fig S3A). Staining with anti‐hGBP1 overlapped with the mCherry‐hGBP1 signal showing that anti‐hGBP1 can detect hGBP1 in its active, membrane‐associated state and indicating that hGBP1 fails to associate with PVs in human A549 cells. We stained IFNγ induced MEFs with anti‐hGBP1 to ensure our antibody did not overlap with mGBPs at the PV (Fig S3B). Last, we expressed mCherry‐hGBP1 in A549 cells and failed to observe any association of mCherry‐hGBP1 *Toxoplasma* PVs (data not shown), further corroborating our finding that in striking contrast to mGBP1 and 2, hGBP1 is not recruited to intact vacuolar membranes of intracellular pathogens in infected human cells.

### hGBP1 restricts Toxoplasma, *but not the intravacuolar bacterial pathogens* C. trachomatis and S. typhimurium

**Figure 2:**
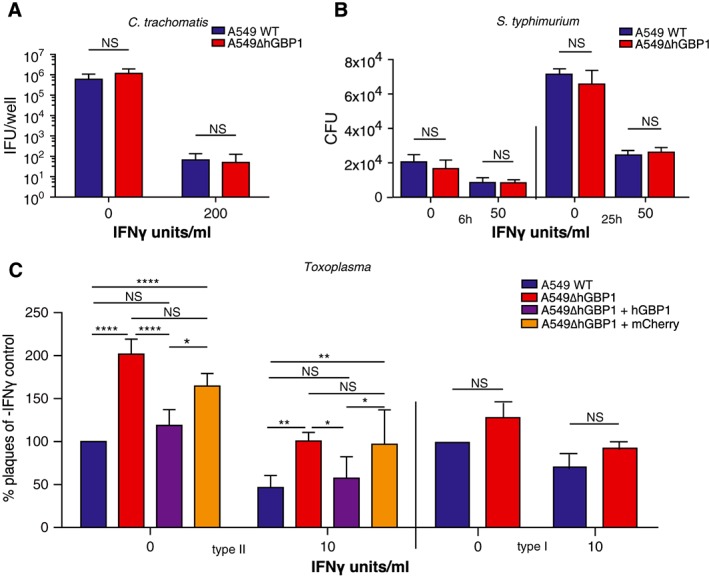
hGBP1 restricts growth of *Toxoplasma gondii* but not *C*. *trachomatis* or *S*. *typhimurium*A. *C*. *trachomatis* IFU assays carried out in A549 cells primed or not with 200 U/ml IFNγ, 40hpi. *N* = 2. Error bars represent standard error of the mean (STEM).B. *S*. *typhimurium* CFU assays carried out in A549 cells primed or not with 50 U/ml IFNγ, 6 and 25hpi. *N* = 3. Significance was analysed by 2way ANOVA.C. Plaque assays showing viability of *Toxoplasma* incubated for 6 h in WT, ∆hGBP1, ∆hGBP1 + hGBP1 or ∆hGBP1 + cherry A549 cells primed or not with 10 U/ml IFNγ before seeding onto HFF. Counts were taken 4 days after HFF infection. *N* = 5, Complementation *N* = 3. Significance was analysed by 2way ANOVA.

As the subcellular location of hGBP1 was not consistent with observations made with mGBPs and the roles they play during intracellular pathogen infection, we were interested to determine whether hGBP1 could still play a role in controlling these particular infections. We assessed the ability of the pathogens to replicate in the absence of hGBP1 in comparison to their wild‐type cells. For *C*. *trachomatis* and *S*. *typhimurium* infections, inclusion forming unit (IFU) or colony forming unit (CFU) assays were employed, respectively, in the presence or absence of IFNγ. Priming cells with IFNγ restricted the replication and growth of both *C*. *trachomatis* and *S*. *typhimurium* (Fig. 2A and B). However, the ability of bacteria to sustain themselves via replication in the presence or absence of immune pressure was the same regardless of the presence of hGBP1 (Fig. 2A and B). We then determined whether *Toxoplasma* replication was influenced by the absence of hGBP1. We compared the capacity of the parasite to replicate and form plaques in fibroblasts after an initial infection in A549 cells primed or not with IFNγ. Type I or type II *Toxoplasma* were incubated in A549 cells for 6 h, a time period too short for replication to occur, meaning potential differences in plaque number would be a result of parasite killing or early restriction. Twice as many type II *Toxoplasma* were able to survive when the infection took place in ∆hGBP1 cells, while type I parasites remained unaffected (Fig. 2C). Intriguingly, this was still true in cells that were not primed with IFNγ. An immunoblot confirmed the presence of hGBP1 even at basal steady state level (Fig. S1B). In accordance, the number of viable *Toxoplasma* parasites was largely reduced to levels seen in parental A549 cells when ∆hGBP1 A549 cells were complemented with hGBP1 (Fig. 2C). Thus hGBP1 does not restrict replication of the bacterial pathogens *C*. *trachomatis* and *S*. *typhimurium*, but does promote restriction of type II *Toxoplasma*.

### hGBP1 mediates early replication of Toxoplasma *infection*

**Figure 3:**
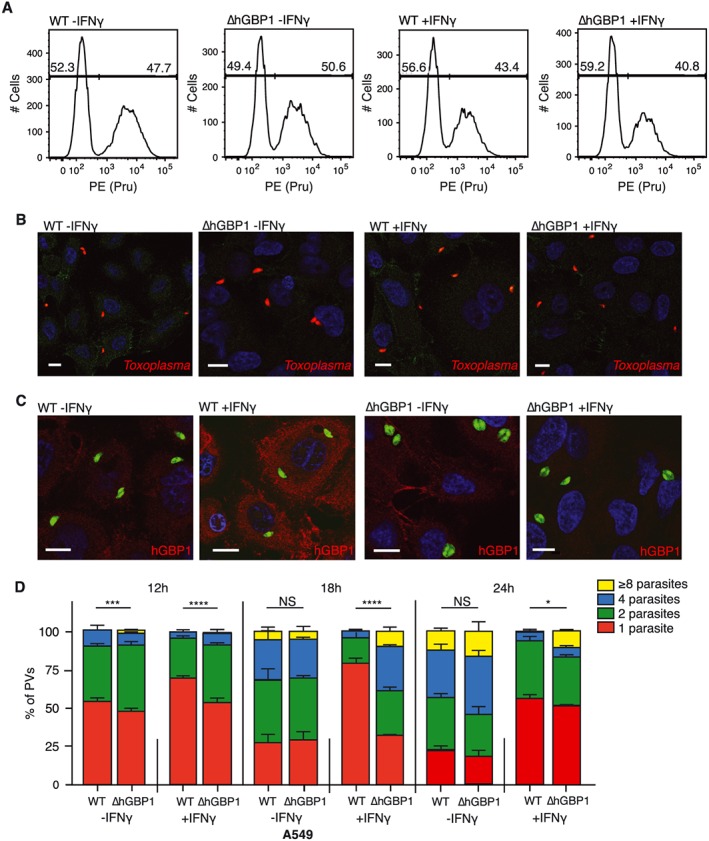
hGBP1 mediates early replication of *Toxoplasma gondii.*A. FACS analysis determined the number of A549 cells that had been invaded by *Toxoplasma* 1hpi. Cells had been primed or not with 10 U/ml IFNγ. Representative of 3 independent experiments.B. Immunofluorescence confocal images taken at 1hpi show the same numbers of parasites enter individual cells.C. Immunofluorescence confocal images of A549 cells primed or not with 10 U/ml IFNγ.D. Replicating parasites were determined by counting the number of parasites within vacuoles at specified times post infection. *N* = 2. Significance was analysed using 2way ANOVA.All scale bars 10 µm.

As we determined that *Toxoplasma* underwent early restriction by hGBP1, our next move was to identify the stage at which parasites were limited. We excluded differential invasion as a reason for the enhanced recovery of parasites from ∆hGBP1 cells. FACS analysis of A549 cells infected with fluorescent parasites showed that regardless of genotype of the cell, type II parasites invaded around 50% of A549 cells when an MOI of 4 was used (Fig. 3A). Immunofluorescence microscopy was used to ensure that individual cells of each genotype were infected with low and equivalent numbers of parasites (Fig. 3B). We used immunofluorescence microscopy to assess the early replication status of *Toxoplasma* in A549 cells. At 24 h post infection (hpi) it was evident that ∆hGBP1 cells contained more vacuoles filled with 2 or more parasites than co‐isogenic wildtype cells did (Fig. 3C). By counting the number of parasites per vacuole over an infection time course, we quantified the replication efficiency of *Toxoplasma* (Fig. 3D). At 12hpi significantly more vacuoles contained parasites that had replicated once in the ∆hGBP1 cells as compared to the wild‐type counterpart, regardless of IFNγ priming. By 18hpi, numbers of vacuoles containing 2 or 4 parasites, indicating 1 or 2 replication cycles, respectively, were significantly increased in the IFNγ primed ∆hGBP1 (Fig. 3D). Finally, counts taken at 24 h show a significantly increased number of vacuoles that hold parasites that have undertaken 3 or more replication cycles in the IFNγ primed ∆hGBP1 A549 cells.

Our observations suggest that hGBP1 can delay the onset of parasite replication. We concluded that type II *Toxoplasma* is restricted early after invasion in A549 cells by a yet unknown mechanism driven by hGBP1 without the protein targeting to the vacuole.

## Discussion

We find that hGBP1 mediates an initial attenuation of type II *Toxoplasma* early post‐infection, without impacting subsequent replication of *Toxoplasma*, *C*. *trachomatis* or *S*. *typhimurium* in human epithelial cells. The observed early *Toxoplasma* attenuation is not because of a defect in parasite invasion in the presence versus absence of hGBP1. Rather, we hypothesize that hGBP1 either mediates early killing or slows down early replication of the parasite, as at 12, 18 and 24hpi the ∆hGBP1 cells contain significantly more parasites that have undergone replication. To our knowledge this is the first report demonstrating that an IFN‐inducible GTPase can restrict growth of an intracellular PV‐resident pathogen independently of any detectable association of the GTPase with the PV itself.

Cell‐autonomous restriction pathways for intracellular pathogens driven by host resistance proteins often see the protein localize to PVs. For example, ubiquitin‐driven autophagy and galectin‐mediated control of *Salmonella* relies on a pathogen‐localization step of ubiquitin and galectin (Perrin *et al*., 2004; Thurston *et al*., 2012). Additionally in mouse, Immunity Related GTPases (IRGs) and GBPs mediate cell‐autonomous killing by directly localizing and disrupting PVs (Martens *et al*., 2005; Ling *et al*., 2006). We clearly define that endogenous hGBP1 is not targeted to *C*. *trachomatis*, *S*. *typhimurium* or *Toxoplasma*. We do, however, show that hGBP1 possesses the ability to recruit to *Toxoplasma* PVs when heterologously expressed in mouse fibroblasts (Fig. S3A). These results suggest that human cells lack a cellular pathway for the delivery of GBPs to PVs that is present in mouse cells. In support of this model, we recently demonstrated that the translocation of mGBP1 and mGBP2 to PVs in mouse cells requires GKS proteins, a subset of IRG protein found in mouse but not in human cells (Haldar *et al*., 2015). Therefore, it appears likely that the deficiency of hGBP1 recruitment to PVs in human cells is due in part to the absence of GKS encoding genes from the human genome.

Conflicting reports attributed hGBPs to have a function in intracellular *C*. *trachomatis*, but not *Toxoplasma* control in non‐hematopoietic cells (Tietzel *et al*., 2009; Ohshima *et al*., 2014). Both reports find hGBPs localized to PVs 24hpi. Reduction of bacterial inclusions upon ectopic expression of hGBP1 and 2 was observed in HeLa cells. Curiously the demonstrated localization of hGBP1 is observed for wild type, GTPase‐deficient and helical‐domain‐only protein. We, and others, have previously shown that GTPase‐activity deficient mouse GBP1 does not recruit to PVs (Kim *et al*., 2011; Virreira Winter *et al*., 2011). It is conceivable that detecting overexpressed hGBP1 with antibody staining presents with different results than detection of endogenous hGBP1. Equally, overexpressed hGBP1 might exert a different effect than endogenous hGBP1 and HeLa cells may present with a different restriction pathway than other epithelial cells.

Ohshima et al. successfully knocked out the entire GBP locus in haploid fibroblast‐like human cancer cells, with the resulting cells showing no defect in IFNγ‐induced cell‐autonomous control of *Toxoplasma* at 24hpi. The early restriction of the parasite by hGBP1 we observe may have been missed or may not exist in fibroblasts. They also demonstrate that a low percentage of *Toxoplasma* PVs were decorated with hGBPs at 6hpi. The staining was carried out using an antibody against hGBP1‐5, so it is conceivable that the protein present at that location comprises another family member(s). In contrast to our findings, hGBP1 has been shown to localize to the bacterial inclusion in THP1 macrophages and restrict *C*. *trachomatis* as demonstrated by shRNA knockdown (Al‐Zeer *et al*., 2013). It remains to be investigated if human GBPs can localize to PVs of other pathogens in macrophages. Combined with our results, this leads us to speculate that hGBP1 may restrict select pathogens in a cell type‐ and localization‐dependent manner.

hGBPs are highly upregulated in all stages of infection with intracellular pathogens and are often found in transcriptional analysis of patient samples. Combined with knowledge acquired from murine studies, it is almost certain that hGBPs have an impact on the control of these infections. We find that hGBP1 plays a role in mediating early restriction of *Toxoplasma* soon after infection without directly localizing to the pathogen. Previous definitions of how this host restriction factor family is acting against vacuolar intracellular pathogens were reliant on the protein being present at the vacuole. Clearly, we have to rethink this rather simplistic assessment as a proxy for potential functions especially for the human GBPs. Early restriction of pathogens directly after invasion is the first step a cell takes to combat the invading foreign agent. These early restriction mechanisms have to act in a rapid and precise manner in order to start the cascade of intracellular defense mechanisms. Because hGBP1 is expressed at steady state level and we observe its defense activity even in absense of interferon priming, it most likely interacts with and directs specific cellular machineries that do not require induction. Future work will elucidate the mechanism by which hGBP1 can mediate early restriction of *Toxoplasma*.

## Experimental procedures

### Cell culture

A549 lung epithelial cells were grown in DMEM with glutamine (Life Technologies) supplemented with 10% FBS and cultured at 37 °C in 5% CO_2_. Where appropriate, cells were stimulated overnight by addition of human IFNγ (R&D Systems) to growth media. Human foreskin fibroblasts (HFF) were maintained in DMEM with glutamine supplemented with 1% FBS and cultured at 37 °C in 5% CO_2._

### Bacteria culture and infection

*C*. *trachomatis* serovar L2 434/Bu containing a GFP expression vector (Wang *et al*., 2011) was propagated in Vero cells. Elementary Bodies (EBs) were purified by sequential density gradients (Saka *et al*., 2011), and MOI was determined for purified EBs through infection of confluent Vero cells. For infections, purified EBs were diluted in cell culture medium (DMEM + FBS), and then added to tissue culture dishes containing cells. Infection was facilitated by spinning for 30 min at 1560 ×g at 10 °C. Finally, infection was allowed to continue to the desired time point at 37 °C and 5% CO2. *S. typhimurium* WT strain 22023S (received from David Holden) was cultured in Luria‐Bertani (LB) broth and grown overnight at 37 °C in a shaking incubator. To allow invasive properties of *S*. *typhimurium*, a further culture was produced in anaerobic conditions, gently shaking for 2 h before infection. An optical density reader was used to measure the absorbance at 600 nm wavelength (OD600). The multiplicity of infection (MOI) was calculated using 1.0164/OD600 = µl containing 1 × 10^6^ bacteria and the bacteria were spun at 1000 rpm for 5 min to synchronously infect cells. At 1hpi, cells were washed with PBS and new DMEM medium was added containing 100 µg/ml gentamycin and 10 mM HEPES. At 2hpi cells were washed twice and 10 µg/ml gentamycin was added into new DMEM medium.

### Parasite culture and infection

*Toxoplasma* expressing luciferase/eGFP or tdTomato (RH type I or Prugniaud type II) were maintained *in vitro* by serial passage on monolayers of HFF cells. For infection, *Toxoplasma* were syringe lysed from HFF cultures and added to cell cultures at an MOI 2 for type I parasites, and MOI 5 for type II parasites. The cultures were centrifuged at 1000 rpm for 5 min to synchronize infection, before being maintained at 37 °C in 5% CO_2._ Where appropriate, parasites were irradiated in HFF cells with 15 000rads.

### Antibodies

A unique sequence for hGBP1 was selected (QDLQTKMRRRKAC) and the peptide conjugated to keyhole limpet haemocynin was ordered from BioMatik Corporation, Canada. Rabbits were inoculated with these peptides, and final bleeds taken after 11 weeks (APS, Cambridge).

### Fixed immunofluorescent microscopy

For *Toxoplasma* and *S*. *typhimurium* infections, A549 cells were plated on coverslips (Thermo Fisher) and cultured and infected as above. The cells were fixed in 3% paraformaldehyde (PFA) and permeabilized in Perm Quench (see Supporting Information) before incubating sequentially with primary and secondary antibodies diluted in PGAS (see Supporting Information) for 1 h at room temperature. Coverslips with cells were washed in PBS, with the final wash containing 1 µg/ml Hoechst. Coverslips were mounted on glass slides with Mowiol 4‐88. Slides were viewed on a Zeiss Axioplan II Epifluorescence microscope or on an SP5‐inverted Confocal microscope and analysed using LAS‐AF software. Images were further formatted using ImageJ software. For Chlamydial microscopy, A549 and MEF cells ectopically overexpressing mCherry‐hGBP1 were infected, as above. At 20hpi, cells were fixed with 4% PFA, before being stained with Hoechst in PBS + 300 mM Glycine. After thorough washing in PBS, slides were mounted with Mowiol containing 0.01% p‐phenylenediamine (PPD). Slides were visualized using a Zeiss 510 Inverted Confocal Microscope, and analysed using Zen Blue software.

### Pathogen viability assays

An IFU assay was used to assess *C*. *trachomatis* replication. WT and hGBP1‐deficient A549 cells were plated to confluence in 12‐well plates and stimulated for approximately 16 h with 200 U/ml human IFNγ. Cells were infected with *C*. *trachomatis*, as above, at an MOI of 1. At 40hpi, bacteria were harvested by lysing cells in water for 10 min at 37 °C with frequent mixing, and 5X sucrose‐phosphate‐glutamic acid buffer (SPG) was added to a final concentration of 1X. Bacteria‐containing lysates were then added to cell culture medium (DMEM + 10% FBS), and 10‐fold serial dilutions were performed. These dilutions were used to infect confluent Vero cells in black‐walled 96‐well plates, as above. At 24hpi, infected Vero cells were fixed and permeabilized for 5 min on ice with ice‐cold Methanol, and blocked with 2% BSA in PBS. Wells were stained with a mouse monoclonal anti‐*Chlamydia* LPS antibody, followed by goat anti‐mouse IgG AlexaFluor 488 and Hoechst. For each well, 10 images were taken on a Zeiss Obzerver.Z1 scope using a 20X objective, and the number of *Chlamydia* inclusions/field was enumerated and averaged across the 10 images. The number of infectious units/well of A549 cells was calculated, taking into account the well area represented by each field and accounting for dilution. For each condition, infections were performed in triplicate (3 wells of A549 cells) for a single experiment. *S*. *typhimurium* CFU assays were performed by infecting cells for desired period of time before washing twice with ice‐cold PBS and lysing with 0.5% Sodium deoxycholate (NaDOC) (Sigma‐Aldrich) in PBS. Each sample was mechanically scraped and pipetted up and down. The lysates were diluted appropriately in LB and spread on a pre‐warmed 10 cm^2^ agar plate, incubated overnight at 37 °C followed by colony counting. Parasite viability was assessed by indirect plaque assay. A549 cells were infected with 300 type I parasites or 600 type II parasites in 24 well plates for the desired length of time before scraping and syringe lysing the cell layer to release the *Toxoplasma*. This suspension was then plated onto confluent HFF in a 24 well plate in serial dilutions of 1:2. This infection was allowed to persist at 37 °C at 5% CO_2_ for 4 days, after which plaques were counted using a microscope.

### Parasite invasion assay using Flow cytometry

A549 cells were infected with irradiated type II Pru parasites for 1 h before washing twice in PBS. Cells were lifted with 2X Trypsin, before quenching in DMEM. The cell pellet was washed twice in PBA (see Supporting Information) before fixing with 4% PFA for 20 min on ice. PBA was added to quench reaction before suspension was centrifuged at 1500 rpm for 3 min at 4 °C, after which cell pellet was resuspended in PBA and analysed on a BD LSR‐II. Results were analysed using FlowJo software.

### Overexpression of mCherry‐hGBP1

The mCherry or mCherry‐hGBP1 plasmids were transfected into A549 ∆hGBP1 or SV40 T‐antigen immortalized Mouse Embryonic Fibroblasts using lipid transfection.

### CRISPR/Cas9 mediated deletion of *Gbp1*

The guide RNA sequence hGBP1 guide1, 5′ cattacacagcctatggtgg 3′ to human *Gbp1* was selected using the optimized CRISPR design site crispr.mit.edu. Oligonucleotides were synthesized (Sigma‐Aldrich) and annealed and cloned into the CRISPR vector p48139 containing a puromycin selection cassette (Ran et al. 2013. pSpCas9 (BB)‐2A‐Puro (pX459) was a gift from Feng Zhang. Addgene plasmid no 48139), using the BbsI restriction site, according to the Addgene CRISPR Genome Engineering Toolbox (Zhang Lab) www.addgene.org/crispr/zhang/. Transfection of A549 cells with hGBP1 guide RNA‐containing p48139 plasmid was performed using FuGENE6 reagent (Promega) according to the manufacturer's instructions. Selection using 2 µg/ml puromycin was commenced 30–36 h post transfection and continued for 48 h. The puromycin was then removed and the cells allowed to recover before selecting individual clones. To confirm disruption of hGBP1, clones were cultured and either lysed with cell lysis buffer containing 1% Triton X100 (Sigma) for SDS‐PAGE and immunoblotting with hGBP1‐specific antibody or lysed with DNAreleasy (Anachem Ltd) for subsequent DNA sequence analysis.

## Author Contributions

ACJ, AP, BC, JC and EMF designed experiments, ACJ, AP, BC and MS performed experiments, BC and SVW generated reagents, ACJ, AP, BC, JC and EMF wrote the manuscript, and JC and EMF supervised the study.

## Supporting information


**Fig. S1**. hGBP1 peptide antibody is specific for use in immunoblots and immunofluorescence.
**Fig. S2**. hGBP1 does not localise to the intracellular pathogen vacuole.
**Fig. S3**. hGBP1 specifically recognises hGBP1 targeted to type II *Toxoplasma* PVs in mouse embryonic fibroblasts.

Supporting info item
