# Supplementary material for: Human GBP1 does not localize to pathogen vacuoles but restricts Toxoplasma gondii
Source: Cell Microbiol. 2016 Mar 10;18(8):1056–64. doi: 10.1111/cmi.12579 (PMC4961618; doi:10.1111/cmi.12579)

Supporting information

Supporting Materials and Methods

Pan-hGBP antibody production

Human GBP1 (hGBP1) genes were cloned into pET28a expression vectors and transformed into BL21 *Escherichia coli*. The crude bacterial lysate was affinity purified on nickel nitrilotriacetic acid agarose (Ni-NTA), anion exchange column and subsequent size exclusion column. Approximately 1mg of hGBP1 was injected to immunise a rabbit for polyclonal antibody production (Covance Inc., Princeton, NJ, USA).

Cherry-hGBP1 construct

Ectopic overexpression of mCherry-tagged hGBP1 was accomplished by cloning the gene into pmCherry-C1 (Clontech) using BglII/SmaI sites. This construct was confirmed by sequencing.

Cherry-hGBP1 expression in Mouse Embryonic Fibroblasts

Cherry-hGBP1 was transfecting into SV40 T-antigen immortalized Mouse Embryonic Fibroblasts (MEFs), and assessing targeting to *Toxoplasma* (Pru). To this end, transfected cells were stimulated for approximately 16h with 200U/ml mouse IFNγ, and infected with GFP expressing *Toxoplasma* for 1h. Cells were then fixed, as above, and stained with Hoechst and α-hGBP1 antibody (above). Finally, results were visualized on a Zeiss 510 inverted confocal microscope.

3T3 fibroblasts expressing GFP- or TAP-tagged hGBPs

The GeneSwitch expression system (Invitrogen) was used to overexpress hGBPs in 3T3 cells. hGBP 1-7 sequences were amplified by PCR and cloned into pGene plasmids modified to contain either GFP tag or TAP (6 x His and 3 x FLAG) tag N-terminal to the hGBPs. 3T3 cells were transfected with pSwitch plasmid using TransIT 3T3 transfection reagent (Mirus) and selected using hygromycin B to maintain the plasmid. pGene plasmids containing each hGBP were then transfected into the 3T3 cells containing the pSwitch plasmid and selected using zeocin. The hormone mifepristone was used to drive the expression of the tagged hGBP protein. Primers used to amplify hGBPs were:

hGBP1f 5'AAGAACTAGATCTATGGCATCAGAGATCCACATGACAG3';

hGBP1r 5'CGGCCGCCCGGGTTAGCTTATGGTACATGCCTTTCGTCG3';

hGBP2f 5'AAGAACTAGATCTATGGCTCCAGAGATCAACTTGCC3';

hGBP2r 5'CGGCCGCCCGGGTTAGAGTATGTTACATATTGGCTCCAATGATTTGC3';

hGBP3f 5'GGATCCCATATGGCTCCAGAGATCCACATGACA3';

hGBP3r 5'GGATCCGCGGCCGCTTAGATCTTTAGCTTATGCGACAT3';

hGBP4f 5'GGATCCAGATCTATGGGTGAGAGAACTCTTCACGCT3';

hGBP4r 5'GGATCCGCGGCCGCTTAAATACGTGAGCCAAGATATTTTGT3';

hGBP5f 5'GGATCCCATATGGCTTTAGAGATCCACATGTCA3';

hGBP5r 5'GGATCCGCGGCCGCTTAGAGTAAAACACATGGATCATCG3';

hGBP6f 5'AAGCTTCATATGGAATCTGGACCCAAAATGTTG3';

hGBP6r 5'AAGCTTGCGGCCGCTTAAAAGGGGAGCTTATGCTTT3';

hGBP7f 5’GGATCCCATATGGCATCAGAGATCCACATG3’

hGBP7r 5’GGATCCGCGGCCGCTCAGCTTATAATTTTCTTACCAGGA3’

Immunoblotting

Cells were lysed using 1% Triton-X 100 and protein was quantified using Bradford protein assay. Proteins were separated on SDS-PAGE before being transferred onto nitrocellulose membranes. The membranes were washed for 1h at RT in 5% Blotto before probing sequentially with the appropriate antibody diluted in 5% Blotto for 1h at room temperature, with 3x 5min washes in PBS 0.05% Tween after each incubation. Immobilion Western Chemiluminscent HRP Substrate (Merck Millpore) was added to membrane for 5 minutes at room temperature before developing images using an automated developing machine.

Buffer recipes

Cell lysis buffer: 100mM Tris HCl pH 7.4, 20mM $MgCl_2$, 600mM NaCl, 1% TX-100

Perm Quench: 50mM NH4CL, 0.2% (w/v) saponin in PBS

PGAS: 0.2% (w/v) gelatine, 0.02% (w/v) saponin, 0.02% (w/v) NaN3 in PBS

Blotto: 5% skim milk powder, 0.05% Tween20 in PBS

Supporting Figure Legends

Figure S1. hGBP1 peptide antibody is specific for use in immunoblots and immunofluorescence

A. Immunoblot of α –hGBP1, α –pan hGBP and α-Flag/-GFP on lysate of 3T3 fibroblasts expressing TAP-tagged (72kDa) or GFP-tagged (93kDa) hGBPs.

B. Immunoblot showing hGBP, and specifically hGBP1, expression in A549 lysates induced with IFNγ.

C. Sequence highlighting the region targeted for *Gbp1* disruption in A549 wild type cells and the disrupted area as confirmed by sequencing.

D. Immunoblot of A549ΔhGBP1 lysate with α-hGBP1 and α-pan hGBP. A549 were stimulated overnight with IFNγ.

E. Immunofluorescent confocal images of A549 cells wild type or ΔhGBP1 stained for hGBP1 and induced or not overnight with IFNγ.

All scale bars 10μm.

Figure S2. hGBP1 does not localise to the intracellular pathogen vacuole

A. Immunofluorescent confocal image of unprimed A549 cells expressing mCherry-hGBP1 at 20hpi with *C. trachomatis* vacuoles. N=2.

B. Immunofluorescent confocal image of unprimed A549 cells stained for hGBP1 at 4hpi with *S. typhimurium*. N=3.

C. Immunofluorescent confocal image of unprimed A549 cells stained for hGBP1 and infected for the indicated time points with type I and type II *Toxoplasma*. N=3.

All scale bars 10µm.

Figure S3. hGBP1 specifically recognises hGBP1 targeted to type II *Toxoplasma* PVs in mouse embryonic fibroblasts

A. Immunofluorescent confocal images of MEFs overexpressing mCherry-hGBP1 and stained for hGBP1 (turquoise) induced with IFNγ and infected with type II Pru *Toxoplasma*.

B. Immunofluorescent confocal images of MEFs overexpressing mCherry and stained for hGBP1 (turquoise) induced with IFNγ and infected with type II Pru *Toxoplasma.*

Fig. S1

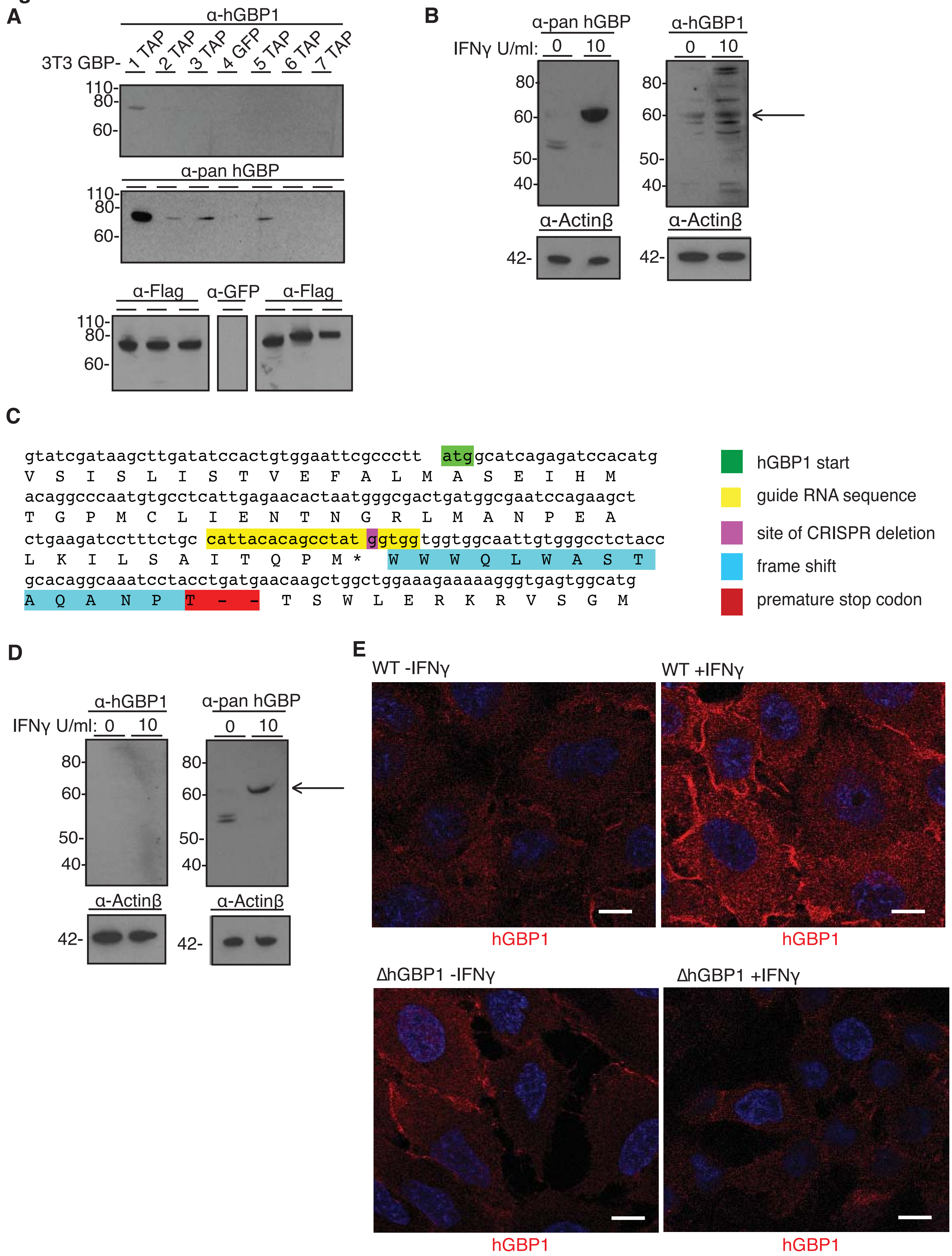
A
α-hGBP1
3T3 GBP- 1 TAP 2 TAP 3 TAP 4 GFP 5 TAP 6 TAP 7 TAP
110-
80-
60-
α-pan hGBP
110-
80-
60-
α-Flag
α-GFP
α-Flag
110-
80-
60-
B
α-pan hGBP
α-hGBP1
IFNγ U/ml: 0 10
0 10
80-
60-
50-
40-
α-Actinβ
42-
C
gtatcgataagcttgatatccactgtggaattcgccctt atggcatcagagatccacatg
V S I S L I S T V E F A L M A S E I H M
acaggcccaatgtgcctcattgagaacactaatgggcgactgatggcgaatccagaagct
T G P M C L I E N T N G R L M A N P E A
ctgaagatcctttctgc cattacacagcctat ggtgg tggtggcaattgtgggcctctacc
L K I L S A I T Q P M * W W W Q L W A S T
gcacaggcaaatcctacctgatgaacaagctggctggaaagaaaaagggtgagtggcatg
A Q A N P T - - T S W L E R K R V S G M
hGBP1 start
guide RNA sequence
site of CRISPR deletion
frame shift
premature stop codon
D
α-hGBP1
α-pan hGBP
IFNγ U/ml: 0 10
0 10
80-
60-
50-
40-
α-Actinβ
42-
E
WT -IFNγ
WT +IFNγ
hGBP1
ΔhGBP1 -IFNγ
ΔhGBP1 +IFNγ
hGBP1

Fig. S2

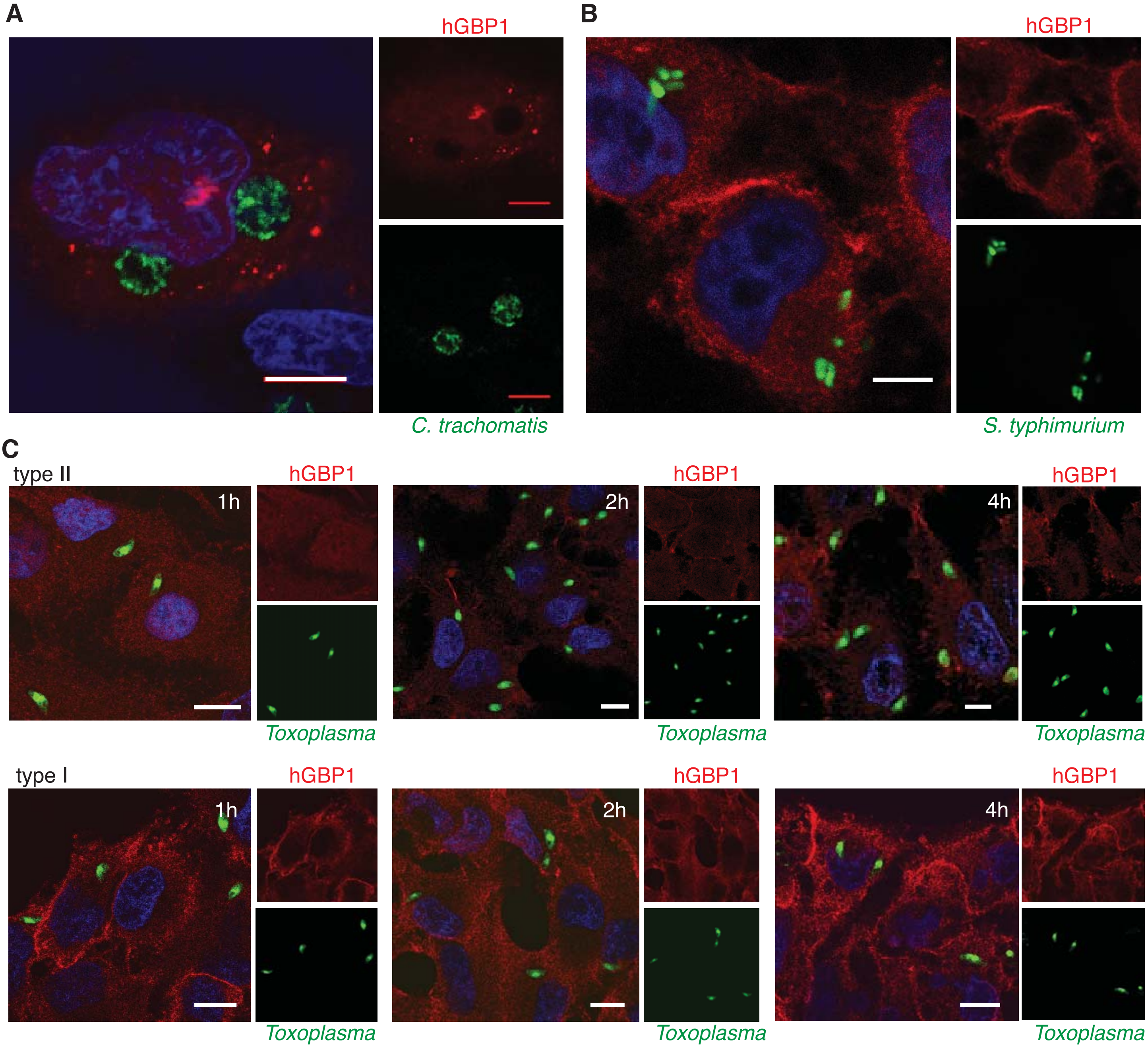
A
hGBP1
C. trachomatis
B
hGBP1
S. typhimurium
C
type II
1h
hGBP1
Toxoplasma
2h
hGBP1
Toxoplasma
4h
hGBP1
Toxoplasma
type I
1h
hGBP1
Toxoplasma
2h
hGBP1
Toxoplasma
4h
hGBP1
Toxoplasma

Fig. S3

A

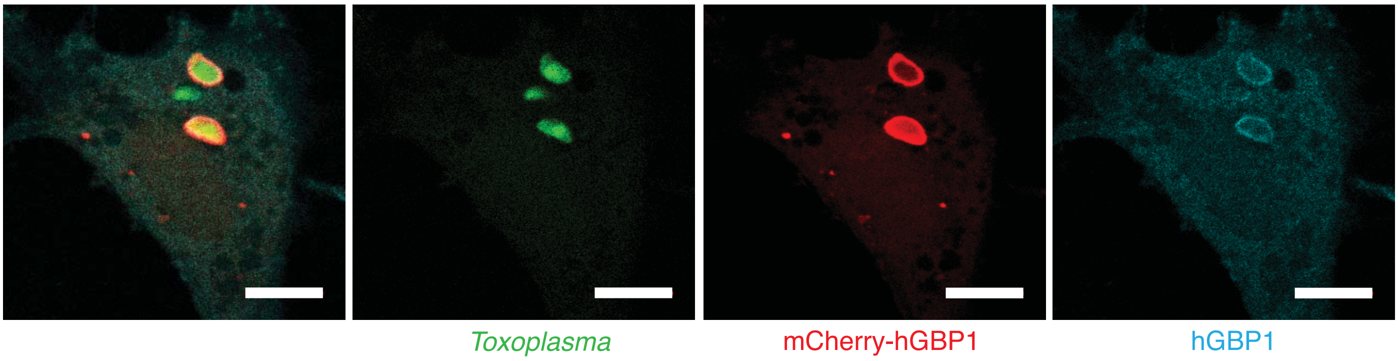

B

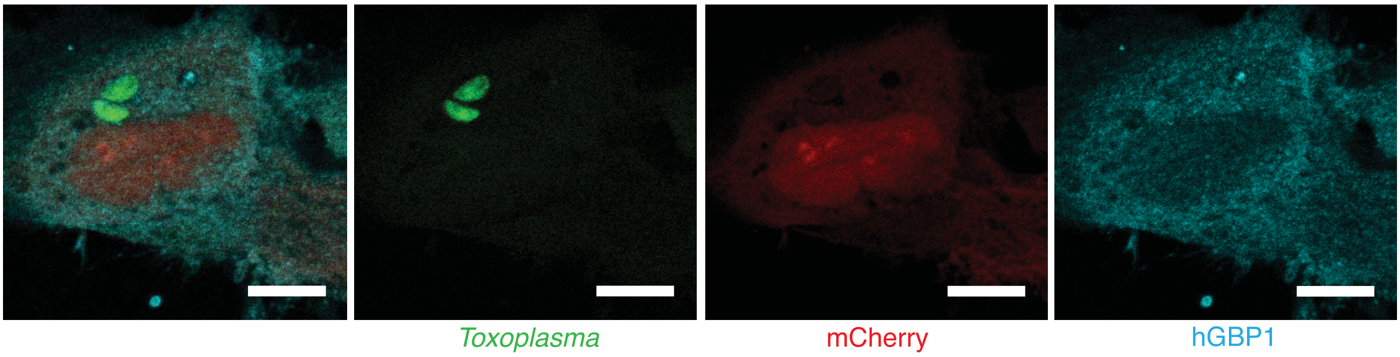

Supplement: Supplementary file 1 — Supporting info item [file CMI-18-1056-s001.pdf]
